# LncRNA MCM3AP-AS1 inhibits cell proliferation in cervical squamous cell carcinoma by down-regulating miRNA-93

**DOI:** 10.1042/BSR20193794

**Published:** 2020-02-07

**Authors:** Liu Lan, Zhishan Liang, Yingxi Zhao, Yuzhen Mo

**Affiliations:** 1 Department of Radiotheragy, The Second Affiliated Hospital of Guangxi University of Science and Technology, Liuzhou, Guangxi 545005, P.R. China; Department of Cardiology, Huadu District People’s Hospital, Southern Medical University, Guangzhou, Guangdong 510800, P.R. China2; 3 Department of Radiotheragy, Guangzhou Red Cross Hospital, Medical College, Jinan University, Guangzhou, Guangdong 510220, P.R. China

**Keywords:** cervical squamous cell carcinoma, MCM3AP-AS1, miR-93, proliferation, survival

## Abstract

**Background:** MCM3AP antisense RNA 1 (MCM3AP-AS1) is characterized as an oncogenic lncRNA in hepatocellular carcinoma and glioblastoma. We analyzed TCGA dataset and observed the down-regulation of MCM3AP-AS1 in cervical squamous cell carcinoma (CSCC). The present study was therefore performed to investigate the role of MCM3AP-AS1 in CSCC.

**Methods:** A total of 64 female patients with CSCC (38–68 years old; mean age: 53.1 ± 6.5 years old) were enrolled in the present study. RT-qPCR was performed to evaluate gene expression. Methylation specific PCR (MSP) was performed to assess the methylation of miR-93 gene after the overexpression and silencing of MCM3AP-AS1. Cell transfections were performed to investigate the interactions between MCM3AP-AS1 and miR-93. Cell proliferation was assessed by CCK-8 assay.

**Results:** The results showed that MCM3AP-AS1 was down-regulated in CSCC and predicted poor survival. The expression levels of MCM3AP-AS1 were inversely correlated with the expression levels of miR-93. Overexpression of MCM3AP-AS1 led to down-regulation of miR-93, while silencing of MCM3AP-AS1 played an opposite role in CSCC cells. Methylation-specific PCR revealed that MCM3AP-AS1 could positively regulate the methylation of miR-93 gene. Cell proliferation analysis showed that overexpression of MCM3AP-AS1 led to reduced proliferation rate of CSCC cells. Silencing of MCM3AP-AS1 played an opposite role and overexpression of miR-93 reduced the effects of overexpressing MCM3AP-AS1.

**Conclusions:** Therefore, MCM3AP-AS1 may inhibit cell proliferation in CSCC by down-regulating miRNA-93.

## Introduction

Cervical cancer is the fourth most common malignancy for both mortality and incidence among all female cancers [1]. In 2018, there were 569,847 new cases of cervical cancer, which accounted for 3.2% of all new cancer cases [2]. A total of 311,365 people died of cervical cancer, accounting for 3.3% of all cancer deaths in the same year [2]. Human papillomavirus (HPV) infection is the major risk factor for cervical cancer [3]. With the advance of HPV screening program and increased understanding of the molecular mechanisms of HPV infection, the incidence and mortality rates of cervical cancer have dropped significantly in many developed countries [3–6]. However, HPV screening rate in developing countries, such as China, is still low [7]. In addition, emerging evidence has shown the presence of HPV-negative cervical cancer, which has unique molecular pathogenesis [8]. Therefore, novel preventative and therapeutic approaches are still of great importance.

It has been well established that HPV infection itself is not sufficient for the development of cervical cancer. The involvement of host factors such as oncogenic proteins are also required [9,10]. Besides, non-coding RNAs (ncRNAs), including long (>200 nt) ncRNAs (lncRNAs) and miRNAs also play critical roles in the development of cervical cancer [11]. Regulation of certain critical ncRNAs in cervical cancer also showed therapeutic potentials [11]. However, the functionality of most ncRNAs remains unknown, which limits the development of targeted anti-cancer therapies [12]. The function of lncRNA MCM3AP antisense RNA 1 (MCM3AP-AS1) has been investigated in several types of cancer, such as liver cancer and glioma [13,14]. We analyzed TCGA dataset and observed altered expression of MCM3AP-AS1 in cervical squamous cell carcinoma (CSCC), a major subtype of cervical cancer. In addition, our preliminary RNA-seq analysis revealed the inverse correlation between MCM3AP-AS1 and miR-93, which is an oncogenic miRNA [15]. The present study was therefore performed to investigate the interactions between MCM3AP-AS1 and miR-93 in CSCC.

## Materials and methods

### Patients and follow-up

The present study passed the review of the Ethics Committee of Guangzhou Red Cross Hospital, and the research has been carried out in accordance with the World Medical Association Declaration of Helsinki. A total of 64 female patients with CSCC (38–68 years; mean age 53.1 ± 6.5 year) were enrolled at aforementioned hospital between June 2012 and June 2014. All the 64 patients signed written informed consent. All patients were diagnosed by histopathological fine needle biopsy. All patients were diagnosed for the first time. No other clinical disorders were diagnosed. No therapies were initiated before the present study. During biopsy, CSCC and non-tumor (3 cm around tumor) tissues were collected under the guidance of MRI. All tissues were confirmed by histopathological exams and were stored in liquid nitrogen before use. All patients were followed-up for 5 years after the admission to record survival conditions. Follow-up was performed in a monthly manner and all patients completed the follow-up.

### Clinical data

The HPV infection situations were determined based on patients’ medical record. There were 50 HPV-positive cases (HPV 16 and 18) and 14 cases of HPV-negative. Based on AJCC staging system, 14, 18, 20 and 12 cases were classified into stage I, II, III and IV, respectively. Treatments were performed including surgical resection, chemotherapies, radiotherapy, or the combination of these.

### CSCC cell lines and cell transfections

Two human CSCC cell lines, C-33A (HPV-negative) and SiHa (HPV positive), were used in the present study. Cells were cultivated in a mixture containing 90% EMEM medium and 10% FBS at 37°C with 5% CO_2_. Cells were harvested at approximately 80% confluence to perform cell transfections. MCM3AP-AS1 expression vector was constructed using pcDNA3.1 vector (Invitrogen) as the backbone. Negative control (NC) siRNA, MCM3AP-AS1 siRNA, NC miRNA and miR-93 mimic were synthesized by Invitrogen. C-33A and SiHa cells were transfected with 10 nM vector, 50 nM siRNA or 50 nM miRNA using lipofectamine 2000 (Invitrogen). Untransfected cells were used as control (C) cells. Negative control (NC) cells were cells transfected with NC miRNA, NC siRNA or empty vector. Cells were harvested at 24 h post-transfection for the following experiments.

### RNA preparations

Ribozol (Sigma-Aldrich) was used to extract total RNAs from CSCC and non-tumor tissues as well as CSCC cells. PureLink miRNA Isolation Kit (Thermo Fisher Scientific) was used to extract miRNAs. DNA-Eraser Genomic DNA Removal Kit (FroggaBio) was used to digest all RNA samples to remove genomic DNA.

### RT-qPCR

PrimeScript RT Reagent Kit (Takara) was used to perform reverse transcriptions (RTs) using total RNA as template. All qPCR reactions were performed using QuantiTect SYBR Green PCR Kit (Qiagen) to measure the expression levels of MCM3AP-AS1 with GAPDH as the endogenous control. The expression levels of mature miR-93 was measured using the All-in-One™ miRNA qRT-PCR Detection Kit (Genecopoeia) with U6 as the endogenous control. All reactions were performed in triplicate manner and the fold changes of expression levels were determined using 2^−ΔΔ*C*^_T_ method.

### Methylation specific PCR (MSP)

Genomic DNA Extraction Kit (ab156900, Abcam) was used to extract genomic DNAs from cells. Genomic DNAs were converted using EZ-96 DNA Methylation-Gold™ Kit (Zymo Research). Taq PCR Master Mix Kit (QIAGEN) was used to perform PCR reactions evaluating methylation of the gene.

### CCK-8 assay

C-33A and SiHa cells were collected at 24 h post-transfection and the effects of transfections on cell proliferation were assessed by CCK-8 assay. Briefly, cells were seeded onto a 96-well plate (4000 cells per well in 0.1 ml medium). Cells were cultivated under aforementioned conditions and were collected every 24 h until 96 h. CCK-8 solution (Sigma-Aldrich) was added into each well to reach the final concentration of 10% at 4 h before cell collection. OD values were measured at 450 nM.

### Statistical analysis

Data from three biological replicates in each experiment were collected and mean values were calculated. All statistical analyses were performed using GraphPad prism 6 software. Paired *t-*test was used to compare differences between CSCC and non-tumor tissues. ANOVA (one-way) combined with Tukey test was used to explore the differences among multiple groups. With the median expression level of MCM3AP-AS1 in CSCC tissues as the cutoff score, patients were divided into high- and low-level groups (*n* = 32). Survival curves were plotted based on the follow-up data. Survival curves were compared between two groups by log-rank test. Pearson’s correlation coefficient was used for correlation analysis. *P* < 0.05 was statistically significant.

## Results

### Down-regulation of MCM3AP-AS1 in CSCC predicted poor survival

**Figure 1:**
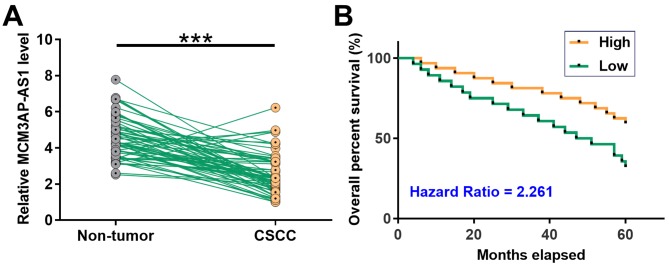
Down-regulation of MCM3AP-AS1 in CSCC predicted poor survival The expression levels of MCM3AP-AS1 in CSCC and non-tumor tissue collected from the 64 CSCC patients were measured by qPCR. PCR reactions were performed three times and data were compared by paired *t* test (**A**). ***, *P* < 0.001. With the median expression level of MCM3AP-AS1 in CSCC as cutoff score, the patients were divided into high and low level groups (*n* = 32). Survival curves were plotted based on follow-up data. Survival curves were compared between two groups by log-rank test (**B**).

Differential expression of MCM3AP-AS1 in CSCC was first analyzed by exploring TCGA dataset. It was observed that expression level of MCM3AP-AS1 was lower in CSCC tissues than that in non-tumor tissues (1.92 vs. 4.88). The expression levels of MCM3AP-AS1 in CSCC and non-tumor tissue collected from the 64 CSCC patients were assessed by qPCR. Paired *t* test showed that, compared with non-tumor tissues, expression levels of MCM3AP-AS1 were significantly lower in CSCC tissues (Figure 1A, *P* < 0.001). It is worth noting that expression levels of MCM3AP-AS1 were not significantly affected by AJCC stages or HPV infections. Survival curve analysis showed that the overall survival rate of patients in low MCM3AP-AS1 level group was significantly lower than that in high MCM3AP-AS1 level group (Figure 1B).

### MiR-93 was up-regulated in CSCC and positively correlated with MCM3AP-AS1

**Figure 2:**
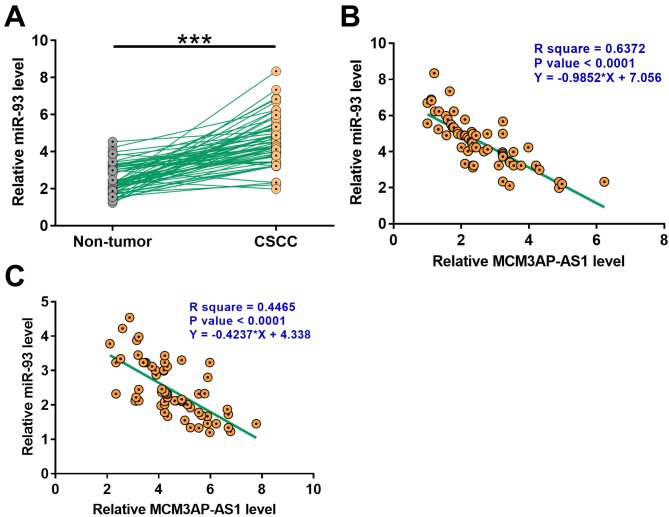
MiR-93 was upregulated in CSCC and positively correlated with MCM3AP-AS1 The expression levels of miR-93 in CSCC and non-tumor tissue collected from the 64 CSCC patients were measured by qPCR. PCR reactions were performed three times and data were compared by paired *t* test (**A**). ***P < 0.001. Pearson’s correlation coefficient was used to analyze the correlation between expression levels of MCM3AP-AS1 and miR-93 across CSCC (**B**) and non-tumor (**C**) tissues.

The expression levels of miR-93 in CSCC and non-tumor tissue collected from the 64 CSCC patients were determined by qPCR. Compared with non-tumor tissues, expression levels of miR-93 were significantly high in CSCC tissues (Figure 1A, *P* < 0.001). Correlation analysis showed that expression levels of miR-93 and MCM3AP-AS1 were significantly and inversely correlated across both CSCC and non-tumor tissue samples (Figure 2).

### MCM3AP-AS1 negatively regulated miR-93 in CSCC tissues

**Figure 3:**
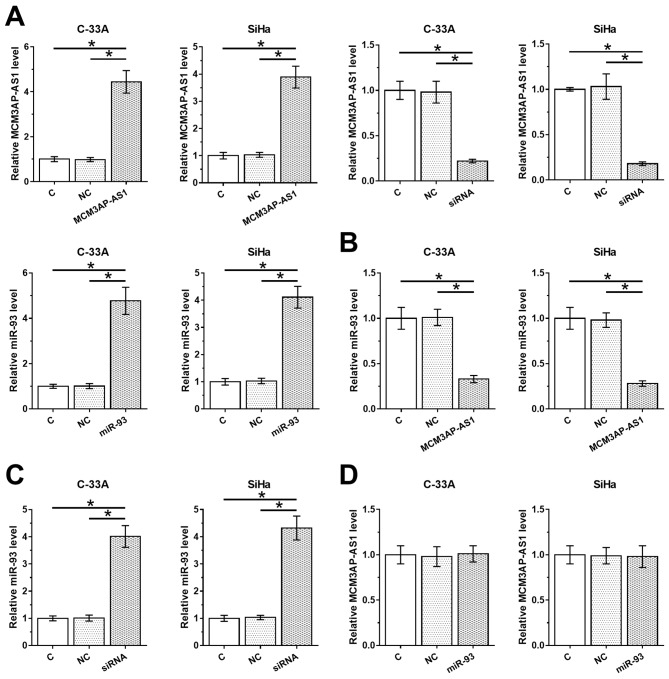
MCM3AP-AS1 negatively regulated miR-93 in CSCC tissues C-33A and SiHa cells were transfected with MCM3AP-AS1 expression vector, MCM3AP-AS1 siRNA or miR-93 mimic. Transfections were confirmed by qPCR at 24 h post-transfection (**A**). The effects of overexpression (**B**) and silencing (**C**) of MCM3AP-AS1 on miR-93 and the effects of overexpressing miR-93 on MCM3AP-AS1 (**D**) were also assessed by qPCR at 24 h post-transfection. Experiments were repeated three times and mean values were presented;*P < 0.05.

To assess the correlations between MCM3AP-AS1 and miR-93, C-33A and SiHa cells were transfected with MCM3AP-AS1 expression vector, MCM3AP-AS1 siRNA or miR-93 mimic. Transfections were confirmed by qPCR at 24 h post-transfection (Figure 3A, *P* < 0.05). Compared with C and NC groups, overexpression of MCM3AP-AS1 led to down=regulation of miR-93 (Figure 3B, *P* < 0.05). In contrast, silencing of MCM3AP-AS1 led to up-regulation of miR-93 (Figure 3C, *P* < 0.05). Moreover, overexpression of miR-93 did not affect the expression of MCM3AP-AS1 (Figure 3D).

### MCM3AP-AS1 positively regulated the methylation of miR-93 gene

**Figure 4:**
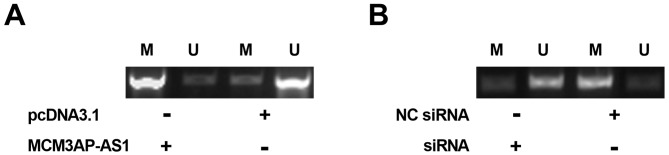
MCM3AP-AS1 positively regulated the methylation of miR-93 gene To explore the mechanisms of the regulation of miR-93 expression by MCM3AP-AS1, the methylation of miR-93 gene after overexpression (**A**) and silencing (**B**) of MCM3AP-AS1 in SiHa cells was analyzed by MSP. Experiments were repeated three times and mean values were presented.

To explore the mechanisms underlying the regulation of miR-93 expression by MCM3AP-AS1, the methylation of miR-93 gene after the overexpression and silencing of MCM3AP-AS1 in SiHa cells were assessed by MSP. It showed that overexpression of MCM3AP-AS1 promoted the methylation of miR-93 gene (Figure 4A). In contrast, silencing of MCM3AP-AS1 suppressed the methylation of miR-93 gene (Figure 4B).

### MCM3AP-AS1 suppressed the proliferation of CSCC cells through miR-93

**Figure 5:**
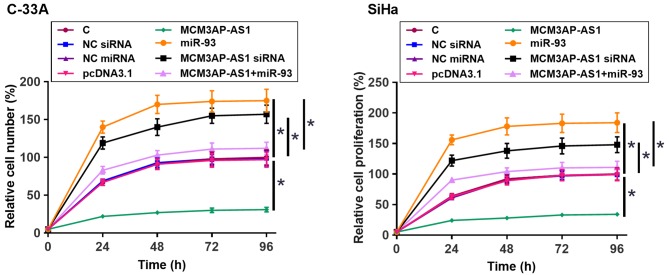
MCM3AP-AS1 suppressed the proliferation of CSCC cells through miR-93 The proliferation of C-33A and SiHa cells transfected with MCM3AP-AS1 or miR-93 overexpression or MCM3AP-AS1 siRNA silencing was analyzed by CCK-8 assay. Experiments were repeated three times and mean values were presented; *P < 0.05.

The proliferation of C-33A and SiHa cells was assessed by CCK-8 assay. Compared with C group, overexpression of MCM3AP-AS1 led to reduced proliferation rate of CSCC cells. Overexpression of MiR-93 and silencing of MCM3AP-AS1 played an opposite role and overexpression of miR-93 reduced the effects of overexpressing MCM3AP-AS1 (Figure 5, *P* < 0.05).

## Discussion

The present study mainly investigated the involvement of MCM3AP-AS1 in CSCC. The results showed that MCM3AP-AS1 was down-regulated in CSCC and suppressed the proliferation of CSCC cells by negatively regulating miR-93.

The functionality of MCM3AP-AS1 has been investigated in several types of cancer [13,14,16]. It has been observed that MCM3AP-AS1 might play different roles in different types of cancer. For instance, MCM3AP-AS1 is up-regulated in liver cancer and can target miR-194-5p/FOXA1 axis to promote cancer growth [13]. In contrast, MCM3AP-AS1 is down-regulated in ovarian cancer and targets miR-28-5p to reduce cancer cell viability and induce cell apoptosis [16]. In the present study, we observed that MCM3AP-AS1 is down-regulated in CSCC and negatively regulated the proliferation of CSCC cells. Therefore, MCM3AP-AS1 is a tumor suppressive lncRNA in CSCC. In addition, the expression of MCM3AP-AS1 was not significantly affected by HPV infection. Therefore, MCM3AP-AS1 may suppress CSCC through HPV-independent pathways.

Although enormous efforts have been made on the treatment of CSCC, the prognosis of this disease is still poor [17]. Our study demonstrated that the low expression levels of MCM3AP-AS1 were significantly associated with the poor survival of CSCC patients. Therefore, evaluating the expression of MCM3AP-AS1 may assist the prognosis of CSCC and therefore provide guidance on the selection of therapeutic approaches and the development of care program. However, our study only investigated the overall survival of CSCC patients. The prognostic value of MCM3AP-AS1 for progression-free survival of CSCC patients remains to be explored.

MiR-93 plays different roles in different types of cancer. MiR-93 is overexpressed in bladder cancer and promotes the invasion and proliferation of cancer cells [15]. In contrast, miR-93 is down-regulated in colon cancer and suppresses the proliferation of cancer cells [18]. In the present study, we observed down-regulation of miR-93 in CSCC and the increased proliferation rate of CSCC cells after the overexpression of miR-93. Therefore, miR-93 is likely an oncogenic miRNA in CSCC.

It has been reported that lncRNAs can regulate the methylation of miRNAs to participate in cancer biology [19]. In the present study, we found that MCM3AP-AS1 could positively regulate the methylation of miR-93. However, regulation of the methylation factors involved in this process remains unclear. We obtained consistent results from two cell lines, while this observation may or may not apply to other CSCC cell lines. Future studies are needed to explore the interaction between MCM3AP-AS and miR-93 in other CSCC cell lines. It is worth noting that the present study is limited by the small number of patients. In addition, we did not include *in vivo* animal CSCC model to investigate the role of MCM3AP-AS and miR-93 in the biology of CSCC. Our future studies will include more patients and animal model experiments to further explore of functionality of MCM3AP-AS and miR-93 in CSCC.

In conclusion, MCM3AP-AS1 is down-regulated in CSCC and may down-regulate miR-93 to suppress cancer cell proliferation.

## Abbreviations

CSCC: cervical squamous cell carcinoma
HPV: human papillomavirus
lncRNA: long ncRNA
MSP: methylation specific PCR
NC: negative control
ncRNA: non-coding RNA
